# The impact of an indigenous proverb on women's mental health: A phenomenological approach

**DOI:** 10.4102/curationis.v38i2.1539

**Published:** 2015-11-23

**Authors:** Salaminah S. Phiri, Fhumulani M. Mulaudzi, Tanya Heyns

**Affiliations:** 1 Department of Nursing Science, University of Pretoria, South Africa

## Abstract

**Background:**

Proverbs and idioms represent cultural and societal beliefs and values inherited from the forefathers. An example is *lebitla la mosadi ke bogadi.* Over many decades African people have used such ancient instructions to counsel women to be resilient in their marriages thus impacting on their mental health.

**Objective:**

The purpose of this article was to explore and describe that proverb and its impact on women's mental health.

**Method:**

Hermeneutic phenomenology was used to explore and describe the proverb and its impact on indigenous women's mental health. The population included married, divorced, widowed and single women who were attending social clubs or networks in the cities of Tshwane and Johannesburg. Snowball and purposive sampling was used to select 57 participants. Five face-to-face interviews and eight focus groups interviews were conducted. Colaizzi's data analysis method was used to analyse data.

**Results:**

Oppression and stigmatisation of women and their families and harmful effects that may result in death were identified as having an impact on women's mental health. Some women shared that they were oppressed in many ways. In addition, they feared stigmatisation should they wish to divorce. They constantly lived in fear of being harmed or killed by their spouses.

**Conclusion:**

There was a need for nurses to develop awareness regarding cultural issues so that women are better served in primary healthcare settings. Women who are suspected of experiencing abuse, should be screened for abuse so that they can be assisted accordingly.

## Introduction and background

In different cultures across the world, proverbs play an important role in people's lives. These proverbs are used to emphasise a point during discussions and also to define different roles between men and women which implies that there is gender connotation within the meaning and interpretations of proverbs (Olasupo, Kikelomo & Adeniran 2012:11). In Africa, proverbs help us make interpretations of our everyday existence through dialogue and individuals’ collective wisdom which is transmitted from one generation to the other, providing insight into how people live and behave (Hussein 2009:97). Although proverbs recognise that women and men co-exist and also reflect that the relationship between men and women should be founded on equality, it is human nature to dominate one another (Olasupo *et al*. 2012:11). The feeling of feeling superior tends to encourage oppression by using proverbs where women are usually considered inferior to men. When women are oppressed, they may suffer from low self-worth. The aim of this paper is to discuss how the indigenous proverb *lebitla la mosadi ke bogadi* (which roughly translates into ‘a woman's grave is at her husband's place or home’) impacts on women's mental health.

The proverb at the centre of this discussion, *lebitla la mosadi ke bogadi*, is one which suggests that once a woman is married, she must stay with the husband and his family (even if he has died) until her life ends as well. Thus, considering divorce or returning to her father's house (her own family) when marital problems arise or when there is a misunderstanding between a wife and her husband, is taboo (Masenya 1998:83). Therefore, *lebitla la mosadi ke bogadi* may be viewed as gender-biased because it symbolically compels women to persevere in where they live and in negative situations. They are not expected to complain about what is happening to them, and sometimes have to persevere in a depressing situation which may result in stress related conditions such as anxiety and depression. This is supported by Balogun (2010:21) who argues that proverbs violate the rights and dignity of women and are indicators of discrimination against women in the Yoruba culture, in Nigeria.

Hussein (2005:72), who analysed the impact of the symbolic meaning attributed to *lebitla la mosadi ke bogadi* on men and women, argues that African proverbs have a tendency to associate maleness with firmness and supremacy and femaleness with meekness, indignity, and powerlessness. Hussein's interpretation is that the proverb deals with the fate of women trapped within the ideal of maintaining their credibility as good wives who keep on enduring, despite physical and or psychological setbacks. The use of the proverb under study is viewed as predisposing women who are in a subordinate position by virtue of being women, to psychiatric disorders such as depression or other mental health problems if they choose to persevere and be resilient in their marriages.

### Problem statement

The proverb *lebitla la mosadi ke bogadi* is commonly used during premarital counselling. It is a linguistic tool used to encourage women to persevere in their marriages irrespective of how difficult their marital situation is. In a gender-based society, language affects the daily lives of women and men as it is used to express people's behavioural patterns (Ellece 2011:44). Premarital advice is given to the woman to inculcate values of perseverance, patience and sacrifice in a marriage which may lead to psychosomatic disorders (Ellece 2011:46). Such premarital teachings and counselling tend to reinforce men's assumed domination over women and often result in women having to endure dysfunctional marriages, causing them psychological problems. Women often suffer as a result of harmful cultural practices and beliefs that subject them to gender inequality under the disguise of cultural and social expectations (United Nations Children's Fund [Unicef] 2000:6). Gender-biased proverbs have the tendency to subject women to be unable to make emancipatory decisions regarding their own lives, thus leading to low self-esteem and depression. Failure to make informed decisions with regard to one's own health issues has the potential to subject women to emotional stress which may contribute to illness and disease.

### Aim of the study

The aim of the article was to describe the impact of the indigenous proverb *lebitla la mosadi ke bogadi* on women's mental health.

### Trends

Proverbs are considered to provide advice and life lessons on how men and women behave in a society and may have an impact on individuals’ health. Proverbs bring forth cultural and societal expectations because they are ancient and are still valued in current times. Mieder (2007:18) believes that proverbs pass judgement and prescribe what people should do in the future. Mulaudzi (2013:154) also asserts that proverbs are cultural and therefore prescribe values and norms for both men and women.

## Research objective

The objective of this article was to explore and describe the impact of the indigenous proverb *lebitla la mosadi ke bogadi* on women's mental health.

## Definition of key concepts

### Proverb

According to Chilisa (2012:133) a proverb is a tool to describe and express socio-cultural events, a community's behaviour and practices handed down from one generation to another. In this article a proverb refers to idioms or metaphors used to direct women's behaviour in their relationships.

### Indigenous

The concept ‘indigenous’ refers to populations and communities that are aboriginal to a particular geographic area (Dondolo 2005:112). In this article an indigenous proverb refers to the metaphor used to guide and direct indigenous behaviour and traditional practices of indigenous women who belong to black cultures in the cities of Tshwane and Johannesburg.

### Mental health

Mental health refers to the ‘state of being’ in which a person is simultaneously successful at working, loving and resolving conflicts by coping and adjusting to the recurrent stresses of everyday living. Mentally healthy people may at times experience severe distress but are generally able to cope well (Uys & Middleton 2010:834). In this article, mental health refers to a situation where women may not be able to cope and adjust to the stressors they experience because of the use of the indigenous proverb *lebitla la mosadi ke bogadi.*

### Contribution to the field of study

Nurses are stationed at primary health care and emergency centres where abused women often report for services. It is then that nurses should be able to identify those clients who have been exposed to abuse or are at risk of abuse. Nurses should be aware of cultural issues which may also impact on their clients’ health. Therefore the significance of this study is to assist nurses in understanding cultural issues, for example the use of the indigenous proverb which may have a negative or positive impact on women's mental health.

## Research method and design

### Research design

The study was qualitative, and hermeneutic phenomenological designs were used to collect and analyse data. The interpretive mode of enquiry was used following hermeneutic phenomenology to uncover the contextual meaning and interpretations of the indigenous proverb under study and how that impact on woman's mental health. According to Denzin and Lincoln (2011:100), constructivists or interpretivists use hermeneutic methods to plan a research study and execute data collection and analysis process. In this article, data collection was achieved through face-to-face and focus group interviews.

### Data collection methods

The researcher conducted face-to-face individual interviews with five participants followed by eight focus group interviews. These participants were allowed to narrate their stories based on the following research question: ‘How do you understand the meaning and interpretations of the proverb lebitla la mosadi ke bogadi’*?* As an aspect of hermeneutic phenomenology, the five interviews developed more like a conversation with the participants as suggested by Polit and Beck (2012:563). Individual interviews are seen to indicate whether cultural groups shared not just similar experiences but also ways of expressing their voices and other perspectives (Morgan 1997:23). However, the participants suggested they would feel free to discuss the topic as part of a dialogue with other women in different groups. The researcher then decided to use eight focus groups which consisted of four to eight participants per group. Although focus group interviews are not commonly combined within the phenomenological paradigm, a number of nurse researchers such as Jasper, Côte’-Arsenault and Morrison-Beedy used focus groups in their phenomenological studies based on the rationale that focus group interviews encouraged interaction and dialogue amongst participants (Bradbury-Jones, Sambrook & Irvine 2009:666). Palmer *et al*. (2010:100) conducted a study on the use of focus groups in interpretive phenomenology, and found that they allow multiple voices to be heard at one sitting.

### Sample

The sample was a total of five face-to-face interviews and eight focus group interviews comprising of four to eight participants. Snowball and purposive sampling was used to select married, divorced and widowed women to be part of the study. Single women were included upon their request as they indicated that they also had experienced the effects of the proverb under study from their married family members. A total of 57 participants which also included the five individual interviews, comprised of 36 married women: 2 were widowed. Seven participants were divorced. Later 12 single women were included at their own request as the proverb had also impacted on their lives; they voluntarily participated in the study.

### Data analysis

Collaizi's seven steps of data analysis were used (Polit & Beck 2012:566). Data collection and analysis occurred simultaneously. The following data analysis steps were followed:

- All the verbatim transcripts were read repeatedly.
- Protocols were read and significant statements extracted.
- New meanings from significant statements were acquired.
- Formulated meanings were clustered into themes and sub-themes.
- Findings were described and integrated to give meaning.
- Writing and re-writing themes and sub-themes were conducted.
- Validation of the findings was ongoing during data collection as an aspect of member checking through deliberate probing (Polit & Beck 2012:591).

Data were transcribed verbatim and field notes were also collected to capture the participants’ expressions, and how they reacted to the questions including non-verbal cues which could not be captured by the voice recorder.

### Context of the study

The indigenous black women who participated in this study belonged to different ethnic groups and often attended social clubs (societies) at different locations in two cities, namely Tshwane and Johannesburg, for social support. Participants were recruited from these social clubs where they used to attend their monthly meetings. At these social clubs, members meet on a monthly basis and give each other support. At every gathering these women choose a date for the next meeting, preferably when they have been paid as they contribute monthly towards their monthly savings. The researcher made contact at these social gatherings with some women who were willing to participate in the study. They later recommended other women who understood the proverb under discussion well. Gaining access to the research site also facilitated meetings with prominent members of the women's groups and the identification of gatekeepers who assisted to secure appointments with the relevant participants.

Participant were visited at their social clubs where they met at a specific time. They were allowed to choose the most comfortable venue for the interviews. These participants had no problem in choosing the venues because some meetings were held at their respective homes or in a restaurant, whilst others were interviewed at a church retreat where the atmosphere was calm and peaceful. The researcher was not in control of the interview settings as the participants were allowed to freely decide on the place where they felt comfortable to be interviewed at.

## Results

The purpose of this article was to explore and describe the indigenous proverb *lebitla la mosadi ke bogadi* and its impact on women's mental health. For the purpose of this article one main theme, ‘aspects of trapped women’ emerged from the transcribed texts. The results were based on the experiences of participants as shared in their own narratives. They further suggested that the proverb may result in women's oppression, contribute to their stigmatisation and that of their families and may be harmful to their mental health, even leading to death.

### Oppression of women

The results revealed that the proverb emphasises the worth of marriage whilst also highlighting the woman's responsibility to work hard and ensure that the marriage works. These participants experienced the proverb as oppression, exercised in the form of an expectation whereby marriage entails taking care of one's husband as well as his extended family. The following quotes support this finding:

‘Women are told by elder women [*aunts who are married*] that they must love and look after their partners; they are not told that they must have their ‘me’ time, to take care of themselves first, not the other persons first.’ (FG2, P1)

‘This is not fair you understand … You are told that you must take care of all the disabled people in his home and you must respect them irrespective of whether your husband comes home or he does whatever.’ (FG2, P1)

It appears that once married, a man retains his freedom but the woman's role in her new family is to be responsible for taking care of her husband, children and all the other members of his family. Often societal expectations dictate the woman's life, therefore, at times, it is unacceptable for a married woman to do as she wishes or make her own choices. The proverb under discussion is often used as a tool to constantly remind women that, once married, they have no other option than to look after and attend to the needs of their husbands, children and in-laws.

It is society's belief that if a woman is married, she has to prove to her in-laws that she is a hard worker who has been prepared to care for others. Often married women are continuously judged by the way they carry out household chores and how they care for their new families, especially the in-laws. Participants confirmed that the new bride sometimes already starts experiencing stress and anxiety on her wedding day. This was expressed as follows:

‘After the marriage ceremony, relatives don’t leave your marital home immediately after the ceremony. They stay to evaluate your ability to cook, clean houses, and take care of a large number of people. They have to be able to say that the money paid for the lobola [*price, often money or cattle that was paid for the bride*] is was not wasted as you are a hard worker. Some women experience it as causing anxiety because one does know what the in-laws expect in this process.’ (P3)

In African tradition, the concept ‘family’ means the nucleus as well as extended family members. As explained in the above quote, the newly wed *makoti* [bride] is expected to display her caring skills to everyone visiting after the wedding ceremony. She has to show her skills in caring for her new family. She is scrutinised and evaluated to determine whether she was taught and prepared sufficiently for marriage by her parents. The cultural expectation of caring for all family members was viewed as a gender-related oppression because the husband was excluded from that common tradition.

Some of the participants expressed the internalised societal expectations negatively; they experienced feeling of guilt should they fail to care for or perform some chores as expectated by the husband's family. This perception was expressed as follows:

‘Then the women always feel guilty that “I didn’t do the laundry for my husband, I didn’t cook”. They feel guilty you understand …’ (FG2, P2)

The narratives suggest that some women are by nature nurturing human beings. These women are often expected to care for a husband, children and all family members and also perform household chores. They ‘always feel guilty’ if they do not fulfill these nurturing roles. The guilt experienced by them, should they fail to carry out some household chores, sometimes results in a sense of having failed in their lives. This is because the woman believes she has failed because she was not well socialised and groomed by her family. Once such guilt surfaces and is experienced by a woman, she may end up over-exerting herself with the intention of compensating for what she believes she did not or cannot achieve to satisfy others.

### Stigmatisation of women and their families

The participants stated that they were afraid of being stigmised and rejected by their family and society if they left their husbands to return to their homes. This fear of being stigmatised and rejected by their own families was described by a participant as follows:

‘They told me that they do not want anybody who comes back from her in-laws because in their family no one has ever come back home from her marriage, if you do it, you would have done something unusual in the family. We are expecting you to stay and endure all … don’t disappoint us.’ (FG3, P2)

One participant indicated that she did not want to be an embarrassment to her family; it was made clear to her that once she was married she could not go back to her maternal home because nobody in her family had ever divorced. Her family feared that their good reputation would be harmed and discredited by other family members and the community members if their daughter divorced. This is because staying married is a societal expectation for all married women.

Participants also emphasised that their own families would never accept them back should they divorce their husbands or simply leave them and return home. This was expressed as follows:

‘If you go back, your mom will also push you away, you see. And the family members and the community will say, ‘Why do you accept her back?’ because she is not supposed to come back from her in-laws’ place … You feel like you embarrassed them [*own families*] if you go back home, yes, and it's like they have mixed feelings towards you. They feel as if they never taught you manners. Fear of rejection causes a lot of stress. The majority of us are suffering from diseases such as high blood and depression already. Going back is also the same you will end in a mental ward as everyone will isolate you [*participants laugh*].’ (FG3, P 3)

Participants feared rejection and humiliation by their own family members and society should they fail to endure in their marriages. Going back home was linked with embarrassment for one's family because it was morally unacceptable for a married woman to return home because they were taught perseverance, endurance and resilience in marriage. It also appears that the main reason why parents reject their own daughters should they divorce is the fear of being judged. It was clear from the participants’ experiences that fear and stigma had impacted on their physical and psychological health.

Another participant agreed that struggling and suffering at her in-law's home was preferable than going back home. She added:

‘People will talk too much about you and your family. I have to persevere in this family and accept everything that happens because I once reported to my mother and she said to me, ‘Go back my daughter, what will people say in the neighborhood? Remember I received and have used *magadi a batho* the money paid for you as lobola.’ (FG3, P4)

The findings revealed that the participant's mother made it clear that her family had already received and used the lobola paid for her; therefore, going back home would make the community question her mother's morals. For women who experience the same rejection as this participant, there is no other solution than to be resilient and endure. They are not expected to act or respond to challenges in their marriages for fear of embarrassing their parents. Perseverance and endurance in a failing marriage appeared to be significant for women to sustain their marriages for fear of being stigmatised.

One participant, although having experienced problems in her marriage, said she could never go back home to report to her mother because that would be awkward and embarrassing for the whole family. She said:

‘You sometimes feel trapped and hanging in space. You are unhappy in your marital home and at the same time you cannot be accepted back home. Everyone rejects you, the society also reject you.’ (FG4, P1)

Feeling trapped, with nowhere to go because one is rejected by everybody, can certainly leave some psychological scars on women who are forced for years to stay and endure in unhappy marriages. For women who experience the same rejection as this participant, there is no other solution than to endure, which may cause depression. Continued exposure to stressful conditions and failure to improve the situation can lead to the development of psychosocial conditions.

### Harmful effects that may result in death

For some of the women who feel trapped in a relationship, the emotional harm and physical abuse they experience in their marriages can be devastating. Both married and single participants shared that they constantly lived in fear that their partners would hurt or kill them. One participant shared the following experience:

‘I told my brother who is a lawyer that I am going to file for a divorce and he cautioned me against it and he said, “This man will kill you my sister, we are not saying don’t do it and we all know that you are not happy but the only thing is that you will die and leave your children.”’ (FG1, P1)

In this scenario, the participant found herself in a life-threatening situation. The irony in this situation was that her brother, although in the legal profession himself, cautioned her against leaving the marriage instead of giving her legal advice regarding divorce procedure. She, therefore, decided to be resilient and move on with her life although she realised she was running the risk of being seriously hurt or killed by her husband. This made the participant live in constant fear, tolerating the hurtful and hostile environment. She had signs of anxiety and stress on her face and weariness in her eyes. Living in a dysfunctional relationship can lead to depression and mental or physical stress.

According to another participant, in modern times women are still vulnerable in an abusive relationship, specifically those who have no money of their own and are unemployed. She explained:

‘Women are not safe these days because when men feel that you are irritating them or they are [*have had*] enough with you, they will tell you that you think you are clever these days and they will kill you … Poor women do not have anywhere to go.’ (FG1, 2)

The participant explained how vulnerable women who live and persevere in abusive relationships live. At times some women are exposed to the danger of being killed at any moment for being ‘irritating’ or accused of thinking they are ‘clever’. Abused women sometimes feel trapped, and live in constant fear in their relationships. Living in constant fear brings about anger and depression, which can cause harm to a woman's health because it is life threatening.

Another participant told the focus group the tragic circumstances surrounding her sister's death:

‘Initially, my mother did not want my sister to be married because she was still busy with her career but both her parents-in-law to be insisted that their son wanted to marry her because they all loved her. After marrying her, he started to be abusive and my mother told my sister to persevere. But things became worse and one day she came back home and decided she was going to leave this man and she asked my parents to give him his lobola back because she said, “I want this man to see that I am out of his life”. My parents and my sister went to have a meeting at her in-laws house. Her husband refused the *magadi* lobola he said the money must be given to the church. The day her husband got back his *magadi* was the day my sister met her death because he stabbed her to death while walking in the street and hanged himself thereafter. So that thing affected all of us emotionally and no one wants to be married since then including myself.’ (FG1, P3).

Painful emotions were observed in the participant's voice as she related the events. She explained that the family members lived in permanent fear of being married or being in a relationship. What evoked painful emotions for this participant was talking about the feeling that her partner could hurt or kill her. Her sister had persevered and thought it was time for her to leave her abuser and be happy. The entire group of participants felt sorry for the woman who had lost her life and shook their heads in disbelief because they felt women were vulnerable and helpless, which is an extremely painful experience. Both the victim's and perpetrator's families lived in grief as a result of their great loss.

In the opinion of the participants, some women fear leaving an abusive relationship because they are afraid of bringing stigma to their families if they divorce, and being killed. In a culture which adheres to *lebitla la mosadi ke bogadi,* women in abusive relationships have few support systems (or in some cases none) from family or the society, as the findings revealed. Being trapped in an abusive relationship sometimes leaves women living in fear and despair which has a direct negative effect on their mental well-being.

## Discussion

The findings revealed that oppression of women was practiced and carried out under the pretext of cultural expectations, as indicated by the use of the proverb under study. This proverb has been considered by some participants, as being oppressive to women because in black cultures married women are encouraged and socialised to care for everyone around them including their husbands, children and in-laws. However, reference to their own care was not considered as part of any socialisation or guidance. These findings are in line with Chitando's (2004:154) affirmation that women, according to tradition, are expected to perform domestic chores and serve all family members including children and the sick. The findings further indicated that married women were culturally expected to care for their husbands, children and the in-laws because it was a societal expectation do so which sometimes caused them stress. A human being is holistic and whenever there is something that is causing dissatisfaction it may lead to stress. According in McEwen 1998 in Read and Grundy (2012:2) repeated daily stressful events over a period may lead to significant physiological changes. Each time the stress response is activated physiological adjustments occur over time and these adjustments lead to accumulated wear and tear of tissues which is called allostatic load (McEwen 1998, in Read & Grundy 2012:2).

According to Mulaudzi (2013:156) the role of a married woman is cooking in the kitchen, rearing children, and taking care of the husband and all members of the family and other relatives. The married woman's responsibility to care for her in-laws and other relatives cannot be challenged as it is embedded in culture and carried through by the use of proverbs such as the one studied. Ali *et al*. (2011:2) agree and add that in carrying out her usual chores, a good woman is also expected to provide income for the family as part of her responsibility.

By implication, women become so involved in the performance of different duties to be carried out for the family members that they neglect themselves sometimes to the point of exhaustion and fatigue. In support of the findings, Rodriguez (1990:22) asserts, women become trapped in a cycle of duties leading to exhaustion, fatigue or extreme stress, and general poor health then ensues. In addition, results revealed that women were made to feel insecure should they fail to fulfil duties prescribed by societal expectations. Therefore, they feel oppressed, unhappy and experience feelings of low self-esteem. Balogun (2013:562) concurs by reporting on the problems of gender inequality in Nigeria where women are made to believe their natural role is to procreate and serve men and that those who were not obeying the rules were considered difficult. To portray the view that women are meant to work like slaves (Balogun 2013:562) report that in China, the alternative name for a woman is ‘slave’, and it is seemingly seen as an ongoing global problem.

The women are expected to conform to societal norms such as caring for all family members without any choice and have no bargaining power and no voice because they are considered inferior to men. This article further revealed married women would often feel inadequate because they did not do chores in the way expected by the husband and in-laws. In support of this finding, Ali *et al*. (2011:3) argue that a ‘good’ wife is one who is seen as a ‘good’ daughter-in-law only if she treats her mother-in-law as her own mother and never complain of being treated badly by other members of the family. Failure to fulfil these expected roles may make a woman feel she is not fit to be a woman and may often lead to mental health problems. Living with guilt may sometimes make these women develop anxieties and depression which could impact on their mental health.

The findings revealed that women were stigmatised in a number of ways. They were socialised into believing they cannot live outside the unwritten laws of culture and proverbs, and if they do they would be stigmatised and ostracised. For example, in this article the fear of being stigmatised brought about a fear of going back home in the event of a failed marriage. In addition, Phelan, Link and Dovidio (2008:262) expand on these findings by asserting that society enforces conformity through social norms, and failure to comply with these norms may lead to prejudices and stigmatisation which also may have negative implications on the affected women's mental health.

Participants said they feared that they or their parents would be stigmatised should they decide to leave their husbands and go back to their parental home and they also feared rejection from their families and society. The proverb studied requires the married woman to stay and die at her in-laws’ place, failing which society would judge her as an outcast because she failed to persevere in marriage. Some married women's parents’ feared the embarrassment an stigma when accepting their divorced daughters home. This finding is supported by Phillips, Moneyham and Tavakoli (2011:359), who argue stigma is the ‘dilemma of being different based on the characteristic perceived to be different from the norm of the society’. In this study participants feared to be judged and devalued by members of families and society.

Stigmatisation may impact on the woman's health as it can lead to stress and anxiety which, in turn, can result in the development of chronic ailments and general poor health. Participants further stated that the majority of women were suffering from high blood pressure and depression which affects their mental status. This is supported by Hatzenbuehler, Phelan and Link (2013:814) who attest that psychological and some behavioural processes are also disrupted by stigma, whereby there could be self-stigmatisation through internalisation of the negative societal perceptions impacting on their mental health.

The findings further indicated that sometimes the maternal family would reject their daughter because they are afraid of being judged by society as having failed in the socialisation of their daughter. Should the rejection happen, the daughter would experience stress caused by parents who reject her, leading to physical and psychological ill health. The inference here is that exposure to constant rejection, stigma and negative views about oneself may cause more harm to abused women and thus lead to stress. According to Logan and Barksdale (2008:206) chronic stress interferes with the function of the brain and can create physiological dysfunction and other chronic illnesses such as mental illness, which requires individuals to enhance social networks and increase self-esteem.

Participants who felt that they were trapped in their marriages indicated they lived in fear in these harmful relationships because they were afraid of being killed. One participant described how unhappy, fearful for her life and how vulnerable she felt in her marriage and could not do anything about it because she feared being murdered. She could not divorce because of fear of being killed and she resorted to her brother who is a lawyer and warned her against the divorce because he also feared she would be killed. This is an indication of how helpless sometimes women are, although there are laws in the country to protect them. Seedat *et al*. (2009:1011) state in their report on violence and injuries in South Africa that half the female victims of homicides are killed by intimate male partners and the high injury rate is driven by gender-based violence. This article further indicates that participants were worried that sometimes when men feel irritated by their partners or wives, they resort to harming or killing them.

Recently, Abayomi, Kolawaole and Olabode (2013:55) referred to media reports in Nigeria which carried horrible stories of men brutally killing their wives because they simply regarded them as their property and in charge of their lives.

Additionally, the results revealed that some young women were afraid of getting married because they feared being killed as was indicated by one participant who lost her sister who was killed by her husband. Mathews *et al*. (2008:552) confirm that South Africa has a high rate of intimate femicide-suicides – the ‘killing of a woman by her intimate partner who commits suicide thereafter’ – which exceeds reported cases elsewhere in the world.

The implication is that the majority of married women tolerate the abuse and live in silent fear of being killed by their spouses. They become afraid for their lives because a violent husband may injure or kill them for a minor argument or even without a reason. Some women who felt afraid to approach a legal team for support and assistance with divorce, often feared that they would be hurt or murdered and they, therefore, decide to stay in their abusive situation. Living in constant fear and being constantly afraid for their personal safety affects women's quality of life, leading to depression, high anxiety, guilt, shame and other health problems such as memory loss and generalised body pains. Bonomi *et al*. (2006:458) asserts that prolonged exposure to inter-partner violence results in higher rates of severe as well as minor depressive symptoms which impact on mental health. In their study on mental health consequences of intimate partner violence in Vhembe district, South Africa, Peltzer *et al*. (2013:548) found that both post traumatic stress disorder and major depression was significantly more common in patients who had a history of domestic violence. According to Green and Ward (2010:122), abused women experience anxiety which should be noticed during consultation with a health care provider and managed accordingly.

## Ethical considerations

Ethical considerations were maintained in this study. The study received approval from the University of Pretoria's Ethics Committee and each participant agreed to participate in the study, signed informed consent forms giving the researcher permission to interview them. Participation was voluntary and participants were informed that they were free to withdraw at any time from the study should they wish to and that there would be no penalty. There were no potential risks for participants, but because the topic was sensitive, the researcher arranged a pastor to assist participants with counselling should the need arise. Participants were informed that they were not going to directly benefit from the study; however, the results of the study would benefit women exposed to the proverb in question and who would be reporting at health care centres and convey their knowledge to nurses who would then be empowered with relevant skills to handle their situation. Confidentiality was observed and no names were revealed in the research reports and scientific journals. Data were also kept in safe custody.

### Trustworthiness

Trustworthiness in this research was maintained through applying all the strategies and criteria for trustworthiness, namely, credibility, transferability, dependability, conformability and authenticity (Krefting 1991:215–216; Polit & Beck 2012:585).

### Implications

The nursing curriculum should include courses on women abuse or prevention of family violence which is perpetrated through culture and language. A basic forensic nursing course should be added to the curriculum so that nurses can assist women who report to the emergency or health centres with abuse. Specific groups of nurses, trained to offer forensic nursing care, should also be trained on how to identify culturally influenced abuse. Women often do not report the abuse, and nurses should be trained to screen for signs of abuse and detect them accordingly. Then they should know how to manage the situation and where to refer the victim to.

## Limitations

This study was conducted in one province only (Gauteng) and the probability of the findings being different if it were conducted in other provinces and more rural settings is not disputed. Although the findings add to the phenomena of the proverb *lebitla la mosadi ke bogadi* they are not transferable to a wider population in other areas because the research was conducted in Gauteng only.

## Recommendations

Nurses should acknowledge that indigenous policies and ideologies do sometimes result in the abuse of women, therefore it should be added to nurses’ priority list in primary health care settings, and accident and emergency units. An urgent need exists for nurses to develop awareness regarding cultural issues so that women are better served in primary health care settings. Awareness of cultural issues as well as signs of abuse, how to handle this in a sensitive way and accordingly refer the women to prevent secondary abuse, is important.

## Conclusion

Proverbs play important roles in peoples’ lives in different cultures and are used to interpret behaviours and everyday existence. The purpose of this article was to explore the impact of the indigenous proverb *lebitla la mosadi ke bogadi* on women's health. Issues which emerged and impacted negatively on women's mental health were ‘oppression of women’ and ‘stigmatisation of women and their families and harmful effects that may result in death’. The implications and recommendations of the findings for the nursing profession have been indicated. The study may also contribute to the empowerment and support of abused women in Gauteng.
